# Editorial: New insights in nucleic acid approaches for vaccine and biologic delivery

**DOI:** 10.3389/fimmu.2026.1802607

**Published:** 2026-03-13

**Authors:** Ebony N. Gary, David B. Weiner

**Affiliations:** The Vaccine and Immunotherapy Center, The Wistar Institute, Philadelphia, PA, United States

**Keywords:** biologic, cancer, DNA vaccine, infectious disease, mRNA vaccine, nucleic acid

**Figure 1:**
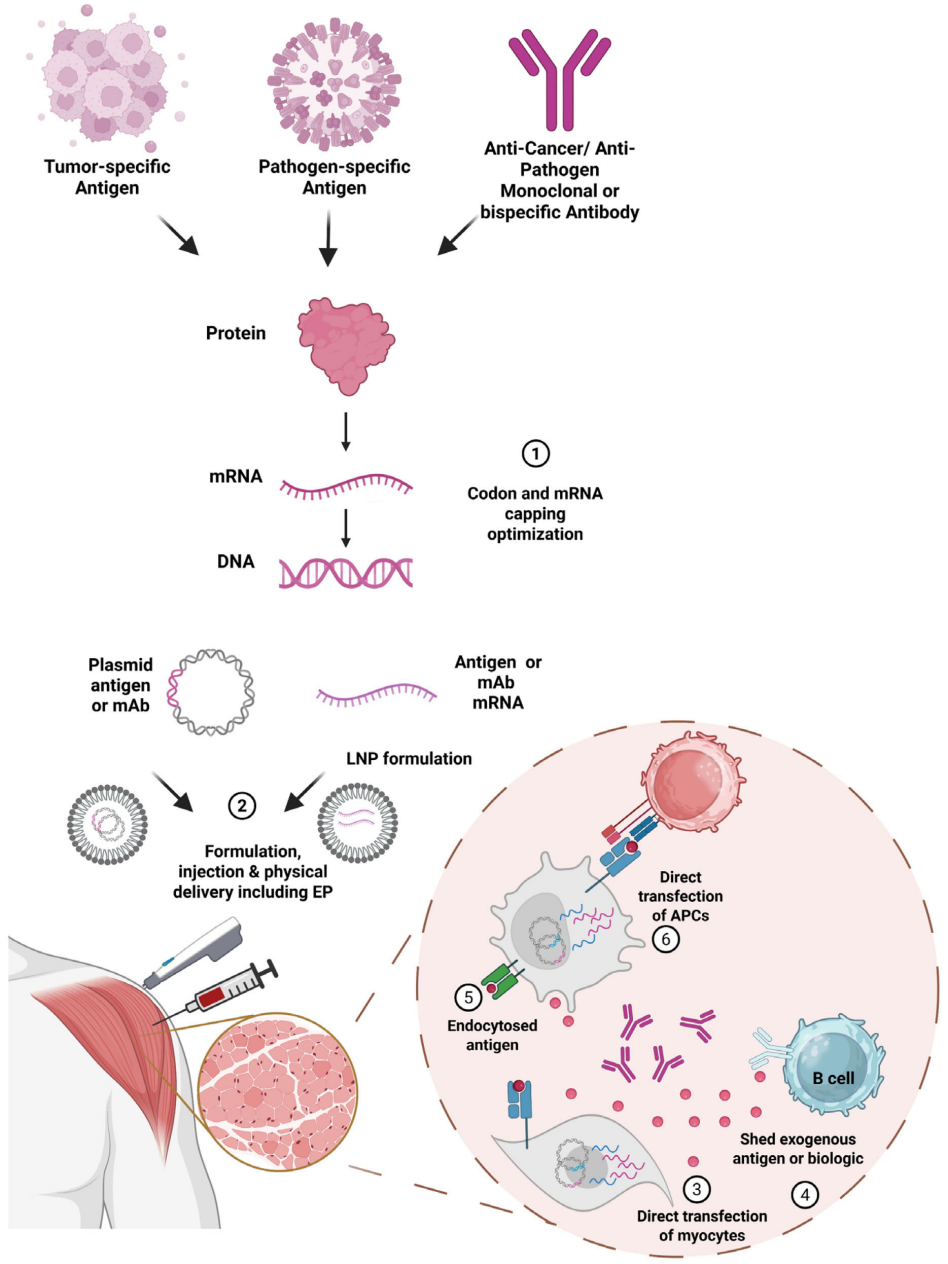
Nucleic acid–based delivery of antigens and biologics for cancer and infectious disease immunotherapy. (1) Tumor-specific antigens, pathogen-specific antigens, and therapeutic monoclonal or bispecific antibodies can be reverse-translated into optimized mRNA or DNA constructs. Codon usage, untranslated regions, and mRNA capping can be optimized to enhance stability and translational efficiency. (2) Antigen or antibody sequences can be formulated as either plasmid DNA or mRNA, including lipid nanoparticle (LNP) or other polymer formulations, and administered via injection with or without physical delivery methods such as electroporation (EP). (3–4) Following administration, nucleic acids may directly transfect myocytes, resulting in the local expression and secretion of antigens or biologics. (5) Secreted products can be shed into the extracellular space and taken up by antigen-presenting cells (APCs) through endocytosis. (6) APCs at the site of injection can be directly transfected to present antigens on MHC I or MHC II.

The last decade has seen an unprecedented increase in the use of nucleic acid platforms, such as DNA and RNA, for vaccine and biologic delivery, culminating in the global deployment of mRNA vaccines during the COVID-19 pandemic. The 2023 Nobel Prize in Physiology or Medicine was awarded for the development of mRNA vaccine technology (1), reflecting the transformative impact of this platform on global health. Nucleic acid delivery approaches offer unparalleled advantages in terms of speed, scalability, and flexibility in antigen design, making them increasingly attractive not only for infectious diseases but also for cancer immunotherapy, autoimmune disorders, rare diseases, and therapeutic delivery. This Research Topic was launched to critically examine the current landscape and future directions of nucleic acid-based approaches for vaccine and biologic delivery. The contributions to this Research Topic include original research, comprehensive reviews, and clinical and preclinical studies, providing both mechanistic insight and translational support for nucleic acid based vaccines and therapeutics (**Figure 1**).

Several articles in this Research Topic highlight advances in RNA vaccine optimization. For instance, Sun et al. explore a new frontier in RNA engineering by demonstrating the use of group IIC self-splicing introns for the development of circular RNA vaccines, with promising results in a mouse model of RSV. Wang et al. focus on optimizing mRNA-LNP delivery using an intradermal, needle-free injection system to induce robust cellular and humoral responses. Two contributions from ST Pharm Co. present a novel mRNA capping strategy (SmartCap^®^), which enhances mRNA stability and protein expression. Choi et al. describe the capping library screening method used to identify the cap, SC101. Next, Kim et al. demonstrate the real-world efficacy of this cap in the context of a SARS-CoV-2 mRNA vaccine candidate. Expanding the use of the RNA platform to bacterial vaccine targets, Roier et al. report the ability of an LNP-formulated mRNA encoding uropathogenic *E. coli* virulence factor FimH to induce functional cellular and humoral responses in mice. 

The DNA landscape is also expertly represented in this Research Topic with preclinical studies of vaccines spanning cancer and infectious disease targets. Helble et al., from the Wistar Institute, describe a DNA-launched nanoparticle vaccine that targets the HPV16 E6 and E7 antigens, showing strong CD8^+^ T-cell responses with translational potential demonstrated in outbred mouse models. Gary et al. report the use of a synthetic consensus approach to generate novel plasmid-encoded influenza nucleoprotein (NP) antigens, which generate robust and durable cellular responses in mice, supporting protection from an H1 influenza challenge. Eisenhauer et al. advance the recently clinically validated DNA-encoded monoclonal antibody (DMAb) approach (2), demonstrating that Fc modification of anti-PcrV DMAbs supports protection against a lethal *Pseudomonas aeruginosa* mouse challenge. In the translational space, studies supporting the use of novel DNA delivery techniques in primates and two clinical trial reports from Inovio Pharmaceuticals underscore the growing translatability of plasmid DNA as a vaccine platform. Sood et al. report on the ability of cationic polymer-formulated plasmid DNA to drive strong and protective cellular and humoral responses in mice and primates. Two clinical studies from Inovio Pharmaceuticals demonstrate the ability of intradermal electroporation (EP) to generate robust and durable responses in humans. Agnes et al. report on the safety, tolerability, and immunogenicity of INO-4700, a DNA vaccine against the MERS-CoV spike glycoprotein. Similarly, Koram et al. reported that INO-4500, a DNA vaccine encoding the Lassa virus (Josiah strain) glycoprotein precursor (GPC), supported strong humoral responses and persistent cellular responses in human patients.

These studies are complemented by several comprehensive reviews. Tadic et al. examine the landscape of anti-angiogenic nucleic acid vaccines and the potential of mRNA vaccine technology in this area. Konopka et al. discuss how aging affects vaccine efficacy and how design considerations specific to the DNA and mRNA platforms can overcome immunosenescence, while Bello et al. provide a thorough summary of nucleic acid vaccine efforts against mosquito-borne flaviviruses, highlighting both current progress and the remaining challenges of achieving broad protection in endemic regions. Complementing these studies, Hojecki et al. provide a comprehensive summary of the use of nucleic acid-encoded adjuvants, termed molecular adjuvants, to enhance vaccine-induced immunity and the recently reported ability of lipid delivery to enhance DNA vaccine-induced responses.

Together, these articles illustrate the depth and breadth of ongoing efforts to harness nucleic acid technologies for diverse applications in disease prevention and treatment. As the field moves beyond pandemic-era urgency to a new phase of innovation and clinical translation, we hope this Research Topic provides both a snapshot of current progress and a foundation for future discoveries.

We thank the authors, reviewers, and journal staff for their contributions to this Research Topic and look forward to continued advances in this rapidly evolving field.
